# Ludwig’s Angina

**Published:** 1917-01

**Authors:** Jno. W. Seybolds

**Affiliations:** Denver, Colo.


### LUDWIG'S ANGINA.

BY JNO. W. SEYBOLDS, D.D.S., DENVER, COLO.

This is a disease that is most likely to be seen first by the family dentist, as the patient realizes that the teeth have something to do with it. The swelling around the jaws and in the mouth gives them the impression.

Ludwig's Angina is not a common disease, but on the contrary is quite rare, so much so, that very few medical men are able to recognize it, yet it is one of the most dangerous to life, the mortality being about sixty per cent.

If the disease is quite well advanced when you first see the patient, which is likely to be (for they do not as a rule seek treatment until forced to), you will notice, as the patient comes in, that there is swelling on one or both sides of the face and neck, that the eyes are dull and the patient's whole attitude is that of one pretty sick. The patient can not open the mouth to any extent; in fact you can barely get your finger in to make an examination and when you do you find the floor of the mouth hard and board-like, with swelling therein tending to force the tongue upward and backward into the pharynx. This makes breathing a little difficult and the patient has a tendency to hold the mouth open.

The swelling on the outside of the face at first is not *red* but gray, hard and immovable, does not pit on pressure, and seems to be attached to the bone. If the swelling is far enough advanced to cause you to lance it, you will find the pus coffee colored and of an offensive odor peculiar to this disease.

The trouble often starts from abscessed teeth, a collection of pus within the floor of the mouth, or a sub-acute swelling which may remain indolent for days, but when it does become active the swelling is very rapid and spreads to the whole floor of the mouth and front of the neck. If the tissues within the mouth rise in a gray roll above the teeth, this indicates a dangerous stage of the disease and resolution is about to set in or has set in and you will get symptoms of sepsis. If the patient is allowed to go without treatment, death will ensue within a week or ten days.

In treating these cases, I have found the best procedure is to send them to the hospital and put them to bed with strict orders to stay there. Give them a course of calomel to get the bowels open. Have the swelling packed in ice and give the patient mixed infection vaccine hypodermatically until you can get an autogeneous vaccine made up from a smear of their own pus taken from the swelling. And let me say right here. *Don't wait until the swelling points, to lance it,* and don't use a general anesthetic if there is an impediment to the respiration. Make an incision, then use a fine pair of hemostats to burrow to the pus, which you will find deep seated and near the glands. Get good drainage and put in a tube to keep the wound open. Syringe out with lysol solution twice a day. Don't forget to see that the patient is getting nourishment; feed by rectum if they are unable to take it by mouth.

They must get rest and orders should be left to give hypodermics when necessary. Insist that the patient stay in bed and not be allowed to run around the room or ward in bare feet (as I have seen them do), and die.

Remember, you are dealing with a grave case of septicemia, and be careful of your hands.

I use the ice in the first stages in order to see if the swelling can be brought down and to stop pus formation. If it has not helped in ten hours, I immediately get drainage and then use dry dressing with a little piece of gauze fluffed up around the end of drainage tube to take care of the pus and to help drain.

The patient must be watched carefully for signs of oedema of the glottis developing and some one should be within reach to do a tracheotomy instantly, as these cases die within two minutes.

The foregoing treatment has proved very successful in our hands, and we have not lost a case in the last two years.—*Dental Digest.*

